# P30 A mysterious case of lymphadenopathy, joint pain and balance disturbance

**DOI:** 10.1093/rap/rkae117.061

**Published:** 2024-11-05

**Authors:** Meghna Prabhakar, Marchesa Ataide-Da Costa, Spencer Ellis

**Affiliations:** East and North Hertfordshire NHS Trust, Stevenage, United Kingdom; East and North Hertfordshire NHS Trust, Stevenage, United Kingdom; East and North Hertfordshire NHS Trust, Stevenage, United Kingdom; BSR Education Committee Member, London, United Kingdom

## Abstract

**Introduction:**

A patient presented with a long-standing history of double vision, balance disturbance, widespread lymphadenopathy and joint pain. Sarcoid was suspected due to its multisystem inflammatory effects, non-specific presenting features and insidious onset. Tissue confirmation was sought. However, multiple non-diagnostic lymph node biopsies led the clinical team to reconsider their presumed diagnosis. Finally, after further investigations and multidisciplinary input, a surprising unifying diagnosis emerged when syphilis serology was requested.

**Case description:**

A 51-year-old man attended the Emergency Department with a two-month history of double vision. Prior to this, he described a year-long history of dry cough, arthralgia, myalgia, pins and needles in his hands bilaterally, weight loss, persistent headaches and balance disturbance. There was no travel or sexual history documented. On examination there was evidence of palpable lymphadenopathy, small papules on his forearms and trunk, bilateral wrist tenderness with limited swelling. His chest was clear and muscle power was preserved.

On further assessment, he was found to have raised protein on cerebrospinal fluid, low vitamin D, raised alkaline phosphatase and alanine transaminase with normal liver imaging and an elevated serum angiotensin converting enzyme (ACE). Other investigations included serum electrophoresis, liver auto-antibodies, paraneoplastic antineuronal antibody screen, nerve conduction studies and hand x-rays which were all unremarkable. In the context of widespread lymphadenopathy, a working diagnosis of sarcoid was considered however lymphoma needed to be excluded. Two lymph node biopsies and, subsequently, a surgical excision was conducted. The histology indicated follicular hyperplasia without features of malignancy, lymphoma or sarcoid.

On follow-up, his outpatient blood tests normalised including the serum ACE and acute phase markers. He also reported improvement of lymphadenopathy and arthralgia although his balance disturbance remained. A Romberg’s test was noted to be positive.

Initial management had included vitamin D replacement and bilateral wrist splints for presumed carpel tunnel syndrome. Given the inconclusive tissue diagnosis coupled with a degree of spontaneous clinical improvement, steroids were not initiated despite the suspicion of sarcoid. Instead, syphilis serology was requested and found to be positive thus revealing a unifying diagnosis for this mysterious case.

**Discussion:**

Syphilis is the clinical manifestation of infection with spirochaete Treponema Palladium (T. Palladium) often transmitted through sexual contact or via vertical transmission. Multisystem involvement is owed to its ability to rapidly invade and infect endothelial tissue, resulting in vasculitis and generalised signs and symptoms as it progresses through its primary, secondary and tertiary phases. It has therefore been given the moniker the “great masquerader”, where it can mimic other multisystem diseases such as sarcoid. Musculoskeletal manifestations of syphilis can present with articular and periarticular pathologies as seen in other inflammatory arthropathies. Where polyarthalgia is a common form of presentation, inflammatory arthritis, periostitis, tensosynovitis, myositis and osteomyelitis have been reported in syphilis. Syphilis can remain latent until the tertiary stage at which point syphilitic arthritis may also manifest for example as a neuropathic joint. This may occur years after initial exposure.

Although not seen here, granuloma formation is commonly seen in tertiary syphilis and can also occur in secondary stages, thus further imitating sarcoidosis where granuloma formation is a defining feature. The mainstay of syphilis eradication is treatment with penicillin antibiotics, often with curative results. In contrast, sarcoid is a chronic non-curable condition where steroids and immunomodulatory agents are used to limit disease progression.

Our patient presented with nonspecific symptoms over a year-long period with follicular hyperplasia on lymph node biopsy, a non-specific histological finding with a wide range of differentials such as malignancy, infection and autoimmune disease. The definitive diagnosis of syphilis was made on positive serology. With the prevalence of syphilis in England rising (9% increase in new cases from 2022 to 2023), it is increasingly important for clinicians to consider it in their workup. Importantly, this case reinforced the need for a comprehensive travel and sexual history when presented with a complex multisystem disease.

**Key learning points:**

• Due to its multi-organ involvement and non-specific symptoms, syphilis commonly mimics other diseases. This case highlights the need for a broad differential workup when faced with multisystem disease, particularly when clinical findings are discordant with seen pathology, as seen here where two sequential lymph node biopsies did not support the presumed diagnosis of sarcoid. In the instance of nonspecific symptoms, comprehensive clinical assessment with sexual and travel histories are also advisable, but it should be noted that the sexual history may not always be reliable.

• As the incidence of syphilis rises across the UK, it is also important for clinicians to consider this disease within their workup, especially as detection via serological testing and treatment is relatively straightforward and can often yield positive outcomes.

